# Variation and conservation implications of the effectiveness of anti-bear interventions

**DOI:** 10.1038/s41598-020-72343-6

**Published:** 2020-09-18

**Authors:** Igor Khorozyan, Matthias Waltert

**Affiliations:** Department of Conservation Biology, Georg-August-Universität Göttingen, Bürgerstr. 50, 37073 Göttingen, Germany

**Keywords:** Conservation biology, Ecology, Zoology

## Abstract

Human-bear conflicts triggered by nuisance behaviour in public places and damage to livestock, crops, beehives and trees are among the main threats to bear populations globally. The effectiveness of interventions used to minimize bear-caused damage is insufficiently known and comparative reviews are lacking. We conducted a meta-analysis of 77 cases from 48 publications and used the relative risk of damage to compare the effectiveness of non-invasive interventions, invasive management (translocations) and lethal control (shooting) against bears. We show that the most effective interventions are electric fences (95% confidence interval = 79.2–100% reduction in damage), calving control (100%) and livestock replacement (99.8%), but the latter two approaches were applied in only one case each and need more testing. Deterrents varied widely in their effectiveness (13.7–79.5%) and we recommend applying these during the peak periods of damage infliction. We found shooting (− 34.2 to 100%) to have a short-term positive effect with its effectiveness decreasing significantly and linearly over time. We did not find relationships between bear density and intervention effectiveness, possibly due to differences in spatial scales at which they were measured (large scales for densities and local fine scales for effectiveness). We appeal for more effectiveness studies and their scientific publishing in regard to under-represented conflict species and regions.

## Introduction

Bears (Ursidae) represent a small family of highly recognizable large mammals acting as cultural and conservation flagships, umbrellas for land management and biodiversity conservation, and as keystone species for their ecosystems^1–4^. While the American black bear (*Ursus americanus*) is flourishing in North America and the brown bear (*U. arctos*) fares well in North America and Russia, the bear species living in circumpolar regions, Latin America and Eurasia outside of Russia are threatened due to habitat loss, range fragmentation and direct persecution^5–8^. In anthropogenic landscapes, negative perception of human-bear co-existence is among the main obstacles to population recovery in areas where bears were previously extirpated^9^. As a result, six out of eight recent species of bears are globally threatened what makes their vulnerability level (75%) much higher than that of carnivores (26%) and mammals (21%) in general^10^.

One of the main factors impinging the ecological and flagship status of bears is the socio-economic conflict between humans and bears that kill livestock, destroy crops, commercial tree stands and beehives, exhibit nuisance behaviour in public areas, and even can attack people^5,11^. Except for the giant panda (*Ailuropoda melanoleuca*), all other bear species are subject to such conflicts. This urges scientists and conservationists to seek practical, long-standing and effective ways to minimize human-bear conflicts by protecting assets which can be damaged by bears.

As bears are omnivorous and use different food resources, diverse interventions have been used to protect human assets, i.e. livestock, crops or public places, from these animals. These interventions can be arbitrarily split into non-invasive interventions (aversion, husbandry and non-invasive management), invasive management and lethal control. As described in Table 1, they consist of a number of specific approaches. Aversion is aimed to trigger negative responses by animals with the aid of acoustic, chemical and physical deterrents, or their combinations^12–14^. Visual deterrents, such as scarecrows and fences with flags hanging on ropes or wires known as fladry, are used against other predators^15,16^ but rarely against bears^17^. Husbandry includes traditional and modern practices to protect certain areas from bear-caused damage such as electric fences, enclosures, guarding animals, and their combinations^18–21^. Non-invasive management is an array of techniques attempting to reduce damage without contacting or even seeing target animals. In case of bears, non-invasive management may include, but is not limited to, the isolation of food and garbage, calving control, vegetation care, change of human habits, livestock replacement, and supplemental feeding^22–26^. Invasive management means active manipulations associated with animal captures, handling and posterior monitoring. For bears, the most common technique of invasive management is translocation^27^, but in other species sterilization and shock collars are also used^28,29^. Lethal control is comprised of shooting, trapping and poisoning, of which shooting is the main legal tool against bears^30^. This division of interventions into categories is for practical reasons only and can include techniques based on similar principles, but belonging to different categories. For example, electric fences, shocking devices and shock collars function similarly by repelling animals with strong electric shocks^21,29,31^. However, it seems most logical to assign electric fences to husbandry because they protect areas, shocking devices to physical deterrents as they target individual animals, and shock collars to invasive management as they are fixed on an animal neck.

**Table 1:** The categories and types of interventions used to protect human assets from bears.

Categories	Types	Description
Aversion	Acoustic deterrents	Yelling, aggressive sounds, discharging firearms, freon horns and bell collars
	Chemical deterrents	Pepper spray, commercial repellents and chemicals
	Physical deterrents	Shocking devices, tree trunk barriers, rubber slugs, slingshots, stone throwing and chasing
	Mixed deterrents	Concurrent application of several deterrents
Husbandry	Electric fences	Fences with charged metal wires which produce electric shocks upon a contact
	Enclosures	Closed roofed (sheds) or open-air (corrals) structures to protect assets, usually at night
	Guarding animals	Use of guarding dogs and llamas
	Mixed techniques	Concurrent application of several husbandry techniques
Invasive management	Translocation	Moving culprit bears away from conflict sites
Lethal control	Shooting	Selective or non-selective shooting of bears
Non-invasive management	Calving control	Herd management to shorten the calving period
	Change of human habits	Keeping barbecue stuff out of reach, use of birdfeeders in cold season, and not feeding pets outdoors
	Food/garbage isolation	Food/garbage removal, use of bear-proof bins
	Livestock replacement	Replacement of sheep by cattle
	Supplemental feeding	Provision of carrion, fruits or other food to avert from feeding on human assets
	Vegetation care	Brush clearing, pruning and fruit harvesting

In spite of a plethora of interventions used, little is understood about how effective they are in reducing bear-caused damage. Comparative studies and systematic reviews of the effectiveness of interventions against bears are lacking, even within the most intensively studied species such as American black bear and brown bear. Such studies are very valuable in their own right by allowing to identify successful and unsuccessful interventions and to replicate successful interventions in the same or other species. We are aware of six reviews describing this issue against terrestrial mammalian predators in general, but a rather small sample size of their bear studies precludes from making firm conclusions^32–37^. The authors of study^33^ summarized that hunting and supplemental feeding of brown bears and American black bears did not reduce damage as natural food availability was the best predictor of damage. The study^34^ reported high effectiveness of enclosures against brown bears, moderate effectiveness of herding and ineffectiveness of guarding dogs against American black bears and other predators. It was found that guarding dogs and llamas are effective against brown bears and American black bears^35^. The largest bear samples compiled in studies^32,36^ showed that the effectiveness of interventions varied greatly between study areas and within ursid species, making it hard to reveal species-specific and general patterns. Due to these limitations, so far it was impossible to find the most effective and the least effective interventions against bears and to compare the effectiveness of non-invasive interventions, invasive management and lethal control. It can be expected that the effectiveness of lethal control and invasive management is lower than that of non-invasive interventions because these two interventions are associated with high mortality and new individuals return over time to resume damaging human assets.

Along with limited and mixed information about the effectiveness of anti-bear interventions, even less is known about how it changes with the duration of interventions and the density of bear populations. It is of high practical value to know how long a given intervention can be successfully applied and at what time threshold it becomes ineffective and should be modified or replaced^37^. Time and density effects on intervention effectiveness are very relevant to bears due to easy habituation of these animals to conspecifics, human presence and novelties^38,39^, which may reduce the effectiveness of anti-bear interventions quite quickly. For example, many deterrents are able to embarrass, repel and keep bears away only during a short period of time, usually several months^32,37,40,41^, but the application of some physical deterrents (shocking devices), electric fences and calving control can be effective during up to several years^37^.

The relationships between bear densities and intervention effectiveness are poorly known, but practically important. This is because bear numbers may influence the rates of inflicted damage and thus predetermine whether interventions would be effective or not at a given bear density. Bears tend to cause more damage to human assets at low densities when food is scarce^30,42^ and also at high densities when food is abundant, bear numbers increase and more bears need more food^26,43,44^. Hence, the effectiveness of anti-bear interventions can be lower than expected when hungry bears become persistent and more aggressive in damaging behaviour. As high density may lead to more bears involved in conflicts, it also could increase the demand for bear removal^45^ and affect the effectiveness of removal techniques such as translocation and lethal control.

In this paper, we compiled a global database of intervention effectiveness against bears and studied how it is related to bear species and densities, duration of intervention application, and intervention techniques. We attempted to find and describe the most effective and the least effective interventions against bears. Further, we tested several hypotheses: (1) lethal control and invasive management are less effective than non-invasive interventions due to high mortality and replacement by new individuals which resume damaging human assets; (2) interventions vary in their effectiveness due to the time required for bears to become habituated to interventions and make them ineffective; and (3) intervention effectiveness is reduced at low and high bear densities when bears search for more food.

## Results

We produced a dataset containing 77 cases of intervention effectiveness from 48 source publications (Dataset S1). Table 2 shows the distribution of these cases across intervention categories, intervention types and bear species. The species were biased towards American black bear and brown bear (χ^2^ = 42.737, df = 3, p < 0.001; Table 2) and intervention categories were dominated by aversion and husbandry (χ^2^ = 16.442, df = 4, p = 0.002), but there was no difference between intervention types (χ^2^ = 12.922, df = 11, p = 0.298). Interventions were applied to protect neighbourhood safety (n = 37), small stock (n = 12), beehives (n = 10), mixed assets (n = 7), cattle (n = 5) and tree plantations (n = 5) (χ^2^ = 59.158, df = 5, p < 0.001), and crops (n = 1) which was excluded. An overwhelming majority of cases originated from the USA (n = 49) followed by Canada (n = 11), Norway (n = 5), Japan (n = 4) and other countries pooled (n = 1 from each of Austria, China, Indonesia, Italy, Slovakia, Slovenia, Spain and Sweden) (χ^2^ = 93.584, df = 4, p < 0.001).

**Table 2:** The distribution of 77 cases used in this study to assess the effectiveness of interventions against bears.

Categories of interventions	Types of interventions	American black bear (n = 40)	Asiatic black bear (n = 4)	Brown bear (n = 24)	Polar bear (n = 8)	Sun bear (n = 1)
Aversion (n = 26)	Acoustic deterrents	2		3	5	
	Chemical deterrents	2		1	2	
	Mixed deterrents	4		1		
	Physical deterrents	4	1			1
Husbandry (n = 19)	Electric fences	5	1	3	1	
	Enclosures	1		3		
	Guarding animals	3		1		
	Mixed techniques			1		
Invasive management (n = 11)	Translocation	6		5		
Lethal control (n = 5)	Shooting	3		2		
Non-invasive management (n = 16)	Calving control	1				
	Change of habits	3				
	Food/garbage isolation	3		1		
	Livestock replacement			1		
	Supplemental feeding	2		2		
	Vegetation care	1	2			

We obtained data for all 77 cases, except for bear density (n = 76, missing datum for a sun bear case^12^) and duration of intervention (n = 75, two missing data for American black bear and polar bear^46^).

**Figure 1:**
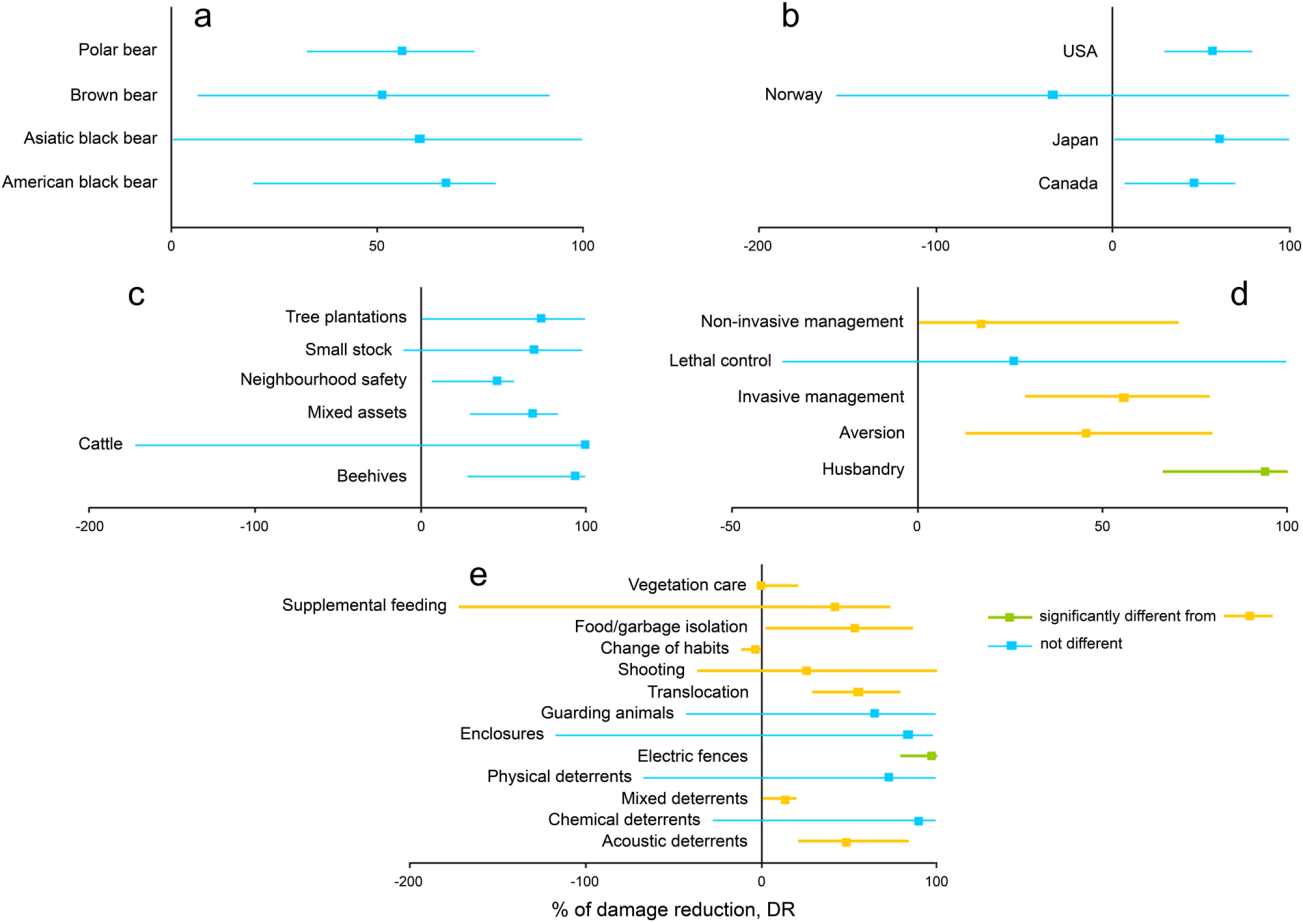
The 95% confidence intervals of the median % of damage reduction across the bear species (**a**), countries (**b**), protected assets (**c**), intervention categories (**d**) and intervention types (**e**) in this study. The samples consisting of only one case are excluded.

In our study, the percentage of damage reduction (DR) differed between intervention categories (Kruskal–Wallis KW χ^2^ = 10.118, df = 4, p = 0.038), but not between intervention types (χ^2^ = 14.954, df = 11, p = 0.185), bear species (χ^2^ = 0.887, df = 3, p = 0.829), countries (χ^2^ = 4.850, df = 4, p = 0.303) and protected assets (χ^2^ = 7.083, df = 5, p = 0.215) (Fig. 1). The effectiveness of husbandry (median DR = 94.2%, 95% CI = 66.7–100%) was significantly higher than that of aversion (45.7%, 13.7–79.5%; Mann–Whitney U = 146.0, p = 0.019), invasive management (55.8%, 30.0–78.8%; U = 49.5, p = 0.017) and non-invasive management (17.3%, 0.3–70.5%; U = 70.0, p = 0.006). The difference between the effectiveness of lethal control (26.1%, − 34.2 to 100%) and other interventions was insignificant (U varied from 20.0 to 56.5, p from 0.161 to 0.836) (Fig. 1). Out of the husbandry interventions, the best were electric fences (97.1%, 79.2–100%) which were significantly more effective than acoustic deterrents (48.4%, 22.7–83.3%; U = 14.5, p = 0.007), mixed deterrents (14.3%, 0.0–20.0%; U = 0.00, p = 0.002), translocation (55.8%, 30.0–78.8%; U = 14.0, p = 0.004), food/garbage isolation (54.1%, 3.8–86.0%; U = 3.0, p = 0.014), supplemental feeding (42.2%, − 171.9 to 73.3%; U = 4.0, p = 0.021) and pooled mixed deterrents (14.3%, 0–20.0%), change of habits (− 3.4%, − 11.1 to 0%), vegetation care (0.3%, 0.3–20.8%), calving control (100%), mixed techniques (97.8%) and livestock replacement (99.8%) (U = 17.5, p = 0.023). Electric fences were marginally more effective than shooting (26.1%, − 34.2 to 100%; U = 9.5, p = 0.050) (Fig. 1). Wherever sample size allowed, these patterns were confirmed also in American black bear which contained most cases (Table 2; Supplementary Data S1).

**Figure 2:**
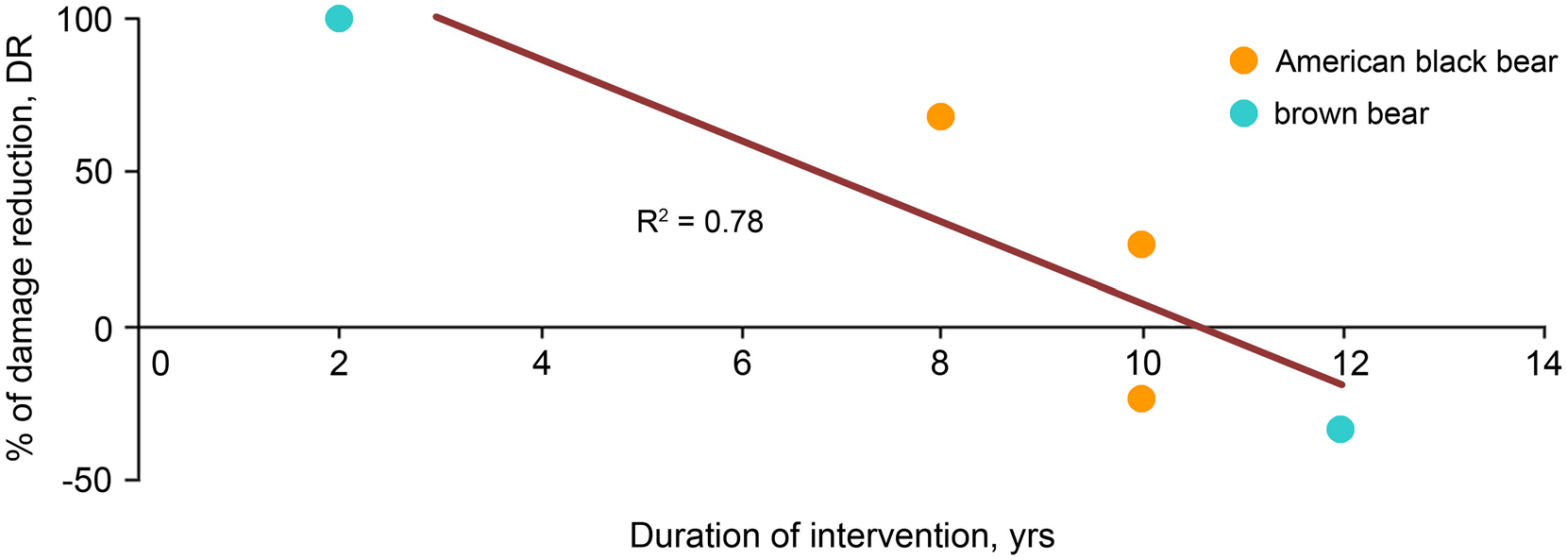
The effect of the duration of intervention on the effectiveness of lethal control (shooting) in American black bears and brown bears. Negative values of the % of damage reduction mean that an intervention is counter-productive by increasing damage.

We found statistically significant effects of duration on DR only in lethal control, i.e. shooting, where DR linearly decreased over time (slope β = − 13.29 ± 4.04, F_1,3_ = 10.841, p = 0.046, R^2^ = 0.78) (Fig. 2). For this equation, DR reaches zero after a duration of 10.54 ± 0.27 years. The mean duration of lethal control was 8.4 ± 1.7 years.

Bear densities differed significantly between bear species (KW χ^2^ = 46.770, df = 3, p < 0.001) and countries (χ^2^ = 20.719, df = 4, p < 0.001; Supplementary Data S2). We did not find significant effects of bear density on DR across intervention categories and bear species (p > 0.05 for all combinations).

## Discussion

Our study has demonstrated that husbandry interventions, especially electric fences, are most effective in protecting assets from bears (Fig. 1). This result appears not to depend on bear species, densities, protected assets and countries involved. In many cases, electric fences may reduce bear-caused losses to beehives, crops and livestock by as much as 80–100% during a period from two months to three years^20,21,47–49^. Possibly, this period can be even longer but studies are often scheduled to finish before the effectiveness of applied interventions begins to decrease. In some cases, however, electric fences were less successful and such cases need careful troubleshooting. The study^50^ reported that records of losses caused by brown bears to apiaries reduced by mere 58%, from 48% of provincial records before electric fences were applied to 20% after electric fences were set up. The authors did not describe factors which might affect the effectiveness, but indicated that they improved the maintenance (grass cutting and moisture control to avoid short-circuit failures, grounding check-up) and materials (aluminum wires, energizers) in use. It was concluded that double fencing can solve the problem in areas where bears become habituated to this intervention^50^. In another study^40^, electric fences reduced nuisance behaviour of polar bears by only 62% because frozen ground and frosting failed to produce high enough voltage (200 kV) essential to reliably affect the dense and thick fur of these Arctic predators. This means that regular monitoring of the performance of electric fences is vital to keep them effective, but its implementation depends on available human and financial resources. An important, but scientifically neglected, issue is the feasibility and practicality of electric fences in challenging environments such as mountains and forests where human activities and bears are most likely to clash.

The other two interventions showing very high effectiveness (95–100% damage reduction) were calving control and livestock replacement, but we found only one case of each in application to bears^23,25^. Still, we find these measures promising and suggest scientists and practitioners to invest more effort into their application and effectiveness estimation. Calving control means shortening the calving season to several months and limiting calf accessibility to open grazing grounds^36^. As calves and other juveniles of livestock constitute most losses, reducing the period of calf availability keeps losses down^25^. This approach requires careful consideration of local seasonality in feed quality, reproductive performance and marketing of meat and dairy products, and takes several years of management adjustments^51^. Livestock replacement aims at substituting frequently killed livestock with livestock that is less likely to be killed by bears or other predators. This can be achieved, *inter alia*, by replacement of sheep by cattle^23^ or heavy sheep breeds by more agile ones^52^, but only the former option was tested in bears. Bears may indeed kill fewer cattle than sheep, making sheep-to-cattle replacement effective in numerical terms, but in terms of financial benefits this approach can be disputable as the market price of a cattle individual is equal to that of several sheep.

Deterrents are reported to be among the least effective interventions against predators because they cause fast habituation and their effectiveness tends to go down over time^37^. Although we found aversion techniques to be significantly less effective than husbandry, this was applicable mostly to acoustic and mixed deterrents vs. electric fences (Fig. 1) and generally deterrents had similar effectiveness to other interventions, except for electric fences. This was caused by high variation of the effectiveness of deterrents in different study cases, with successful applications in some cases and failures in the others. In only 10 out of 26 aversion cases did we find effective applications of deterrents which reduced damage by 50–100%^12–14,31,40,46,53,54^. At the other extreme were 11 cases, in which deterrents reduced damage poorly by less than 20%, did not make a change or even increased damage^41,46,55–59^. Considering variable responses of bears to deterrents and the fact that deterrents remain effective during only several months^37^, we agree with other scientists^32^ and recommend to use these interventions mainly during the peak periods of damage infliction.

Extreme case-specific variability in bear responses and, hence, in effectiveness was found also in other non-invasive interventions (Fig. 1). Guarding dogs and llamas lead to a strong reduction in livestock losses to bears by 60–100%^18,60^, but limitations in study design and environmental conditions may reduce their effectiveness^61^. Isolation of food and garbage through their removal or disposal in special bear-proof bins is often the first solution to get rid of nuisance bears in human neighbourhoods, and it is generally effective by reducing damage by 50–90%^62,63^. However, the study^24^ has concluded that this intervention can make no change if the involved public does not demonstrate responsible behaviour. This is also applicable to other interventions reliant on human behaviour, such as the change of human habits related to animal feeding and storage of food-related stuff^24^. Supplemental feeding can be effective (ca. 70% damage reduction^64,65^) or ineffective and even counter-productive^26,44^, but in the latter studies it was confounded by an increase of bear and livestock numbers that kept damage high in spite of the intervention in place. Vegetation care, such as harvesting fruits from trees and cutting grass and shrubs near trees that can be damaged, is generally unsuccessful and its effectiveness is also influenced by low levels of responsible behaviour^22,24^.

We found partial support for the first hypothesis that lethal control (shooting) and invasive management (translocations) are less effective than non-invasive interventions. In our study, shooting and translocations were significantly less effective than the best intervention, namely electric fences, but the effectiveness of shooting overlapped with all the others due to high variation (Fig. 1). The reasons can be high mortality, non-selective removal when culprit individuals remain unaffected, short distances between capture and release sites for relocated individuals, and replacement of removed individuals by newly coming and returning individuals, which may resume damaging human assets^43,66,67^. These two interventions take years to produce measurable impacts, allowing sufficient timespan for damage levels to recover or even aggravate. We found a negative effect of the duration of interventions on the effectiveness of both shooting and translocations, but only in shooting it took a statistically strong linear effect, in spite of a small sample size (Fig. 2). According to this relationship, lethal control can be effective during up to 10–11 years and then its effectiveness subsides, with a potential to even become counter-productive. Thus, our support for the second hypothesis of time dependence of interventions was also partial as no interventions other than shooting demonstrated significant effects of their duration on effectiveness. The study^53^ claimed a 100% damage reduction when chronic livestock-killing bears were shot, but it lasted only two years compared to 8–12 years in the other shooting effectiveness studies of our sample. Even when shooting is estimated to be effective in reducing bear-caused damage, this relationship becomes confounded by food availability as low damage can be associated with sufficient food, not with a pressure of shooting^30^. During long-term lethal control programs, it can be most practical to shoot bears non-selectively upon encounters. This approach is counter-productive as it may keep damage levels high by targeting mostly innocent individuals, whereas surviving culprits would cause damage as before^67^. Also, shooting within a permitted quota can be ineffective when bear populations increase and offset removals^43^. Overall, in addition to being expensive, ecologically adverse and disliked by the public, shooting is incapable of curbing human-bear conflicts in a long run, but can act only as a short-term solution^68^.

We did not find support for the third hypothesis that the effectiveness of anti-bear interventions decreases at low and high densities of bears. In our study, there was no relationship between intervention effectiveness and density within bear species. Most likely, this is a result of incongruence in spatial scales of inter-related effectiveness and density estimation studies. As bear densities were estimated over large areas and the effectiveness studies were much more localized, in many cases several effectiveness data were juxtaposed with one density datum and so their variation was too high. However, we think that the research of relationships between bear densities and intervention effectiveness is still possible at similar scales on high-density species such as American and Asiatic black bears.

Most information in our study came from American black bear and brown bear, mainly from North America, and much less was available about brown bear in Eurasia, Asiatic black bear and polar bear. Only one case study was obtained for sun bear and no information was found about sloth bear and Andean bear, although human conflicts with these species are common^12,69,70^. We urge for more effectiveness studies and their scientific publishing in regard to under-represented species and regions.

## Methods

### Literature search

We applied several approaches to collect as much information as possible about the effectiveness of interventions against bears. First, we used bear-related publications from the known reviews of the effectiveness of predator-targeted interventions^32–37^. Second, we searched for relevant publications in Web of Science (https://www.webofknowledge.com) in 1970–2019 using the Latin names of seven bear species, except for giant panda (*Ursus arctos*, *Ursus americanus*, *Ursus thibetanus*, *Ursus maritimus*, *Melursus ursinus*, *Helarctos malayanus* and *Tremarctos ornatus*) in combination with eff* which accounts for “effectiveness”, “efficiency”, “efficacy” and “effect”. We did not use the word "bear" in the literature search as it yielded many irrelevant records. Third, we read all issues of the journals Conservation Evidence (https://www.conservationevidence.com, 2004–2019) and Ursus (https://www.bearbiology.org and https://www.bioone.org, 1968–2019). Fourth, we read all issues of the newsletter Carnivore Damage Prevention News (https://www.lcie.org and https://www.medwolf.eu, 2000–2005 and 2014–2018).

Throughout the literature search, we considered only the studies where damage was unequivocally assigned to one bear species, not to several bear species or to several predators including bears, and which were conducted in wild conditions. Further, we excluded the studies which (1) implied the effectiveness from correlation (e.g., more electric fences, less livestock losses) and did not conduct special (quasi-)experimental or comparative research; (2) did not describe interventions; and (3) did not contain sufficient information to measure the effectiveness of interventions. As correlational studies do not consider counterfactuals (controls) to see how damage would reduce without an intervention, they cannot be used in estimation of intervention effectiveness. In several cases, publications were produced at different times by the same researchers in the same study sites, implying temporal autocorrelation of their datasets (Alaska^13,71^, Colorado^18,72^). To secure the independence of input data, from each pair of site-specific publications we used the latest ones because they contained more data. Overall, we included all published studies regardless of their study design, duration and statistical approaches which met the above-mentioned conditions and contained sufficient data to calculate the relative risk of damage (see below).

### Data collection

We compiled a dataset of cases where each case described an effect of a particular type of intervention on the protection of a particular asset from a particular bear species in a site^36,37^. We considered the following assets: neighbourhood safety, cattle, small stock, beehives, mixed assets, tree plantations and crops. We took neighbourhood safety as a comfortable situation when a public place is not affected by nuisance, property damage, attacks on people or pets, disturbance through noise or strewn trash, or other adverse effects caused by bears^24^. Damage to livestock, crops, trees and beehives did not make part of neighbourhood safety and was considered separately. Small stock included sheep only or sheep and goats grazing together, depending on local circumstances^19,26^. Whenever lambs and ewes were considered separately^18,72^, we also took them apart as separate cases. Mixed assets incorporated several assets studied together, such as livestock, beehives and crops^21^, so it was not possible to evaluate the effectiveness of protection of each of these assets individually.

We split interventions to protect assets from bears into the categories of aversion, husbandry, non-invasive management, invasive management and lethal control (Table 1). The categories were based on approaches used to reduce damage and the intervention types included individual interventions within a category. The main approaches used in intervention categories are: aversion—reduction of damage by deterring animals (in our case, bears) from human assets; husbandry—reduction of damage by isolating assets from animals; invasive management—reduction of damage by non-lethal, but invasive (with animal handling) interventions; lethal control—reduction of damage by killing animals; and non-invasive management—reduction of damage by non-lethal, non-invasive (no contact with animals) interventions. Intervention types were proper interventions, e.g. translocations as part of invasive management and shooting as part of lethal control. We considered shooting as an active process of searching and killing bears by guns with or without the use of baits or dogs^67^. Therefore, we did not consider euthanasia of live-trapped bears as shooting. Each category of interventions included at least one type of interventions, in total 16 types, which are described in Table 1 and distributed across the bear species as in Table 2.

Apart from the categorical data of species, assets, intervention categories, intervention types and countries, we also collected continuous data of duration of intervention (years) and bear density (individuals/100 km^2^). Duration covered only the period when an intervention was applied, not the whole period when the study was conducted. For example, study^58^ lasted over one year from Apr 2005 to Jun 2006 but the effectiveness of deterrents was tested only during 145 days when nuisance bears returned or not to original sites. In this case, we took 145 days (= 0.4 year) for duration. In contrast, the effectiveness of long-term interventions such as translocations and shooting was estimated in annual terms and their duration was measured directly in years as a period between the study beginning and end. In order to obtain case-specific data of bear densities fitting our data of intervention effectiveness in space and time, we searched for bear densities in the source publications (see above) or in Google (https://www.google.com) using the search words of bear common name, “densit*”, study area and study period from the source publications. When densities were provided as a range of estimates, we used their average for a particular case. We consulted with Google Maps to ensure that the study areas where density and intervention data came from were spatially matching. We used reports, management plans, PhD dissertations/MSc theses, conference proceedings and scientific publications as reliable sources of bear densities (Dataset S1).

### Data analysis

We quantified the effectiveness of interventions by calculating the percentage of damage reduction (DR) as follows:

$$DR = 100 \times (1 - RR) = 100 \times \left( {1 - \frac{{A/N_{t} }}{{B/N_{c} }}} \right)$$

where RR is the relative risk of damage, A is the metric of damage (e.g., number of livestock individuals killed by predators) with a given intervention, B is the same metric without the intervention, N_t_ is the treatment sample size (e.g., number of livestock exposed to the intervention) and N_c_ is the control sample size (e.g., number of livestock not exposed to the intervention or before the intervention is applied)^34,36,37^. RR represents a ratio of the probability of damage risk with the intervention to the probability of damage risk without the intervention. Interventions are counter-productive at RR > 1, ineffective at RR = 1, effective at RR < 1 and become most effective at RR = 0 when A = 0. When DR turns negative, it means that RR > 1 and that a given intervention increases damage instead of decreasing it.

We conducted the analysis for all bear species and for each species separately. Our continuous data were non-normal and did not fit distributions even after transformations. Therefore, we applied non-parametric Mann–Whitney and Kruskal–Wallis tests to compare DR and bear density across the integer-coded categorical variables. For each categorical variable, we pooled small samples (n ≤ 3) or removed them if small sample was only one and pooling was impossible. We determined the effects of duration of intervention and bear density on DR by scatterplots for each intervention category and bear species, and finding the best-fitting linear or non-linear relationships using the coefficient of determination R^2^ as the effect size^73^. As durations of short-term interventions are more accurate (originally measured in days and transformed by us to years) than those of multi-year interventions (originally measured in years), we separated the effects of durations < 1 year and ≥ 1 year. We did not use scatterplots for intervention types because of small sample size (1–11 cases/type, Table 2). We used one-sample χ^2^ test for frequency comparisons and measured the 95% confidence interval (CI) of the median DR by bootstrapping with 1,000 repetitions in iNZight 3.2.1 (University of Auckland, New Zealand). Standard error (SE) was a measure of variation throughout the paper. We used IBM SPSS 24.0 (IBM Corp., USA) for the statistical analysis, unless otherwise indicated.

## Supplementary information

Supplementary file 1

Supplementary file 2

## Data Availability

The original Dataset S1 collected for this study is available within the Dryad Digital Repository at 10.5061/dryad.2jm63xsmq.
