# Supplementary material for: Variation and conservation implications of the effectiveness of anti-bear interventions
Source: Sci Rep. 2020 Sep 18;10:15341. doi: 10.1038/s41598-020-72343-6 (PMC7501236; doi:10.1038/s41598-020-72343-6)
Supplement: Supplementary file 1 [file 41598_2020_72343_MOESM1_ESM.pdf]

Variation and conservation implications of the effectiveness of anti-bear interventions

Igor Khorozyan, Matthias Waltert

Department of Conservation Biology, Georg-August-Universität Göttingen, Bürgerstr. 50, Göttingen 37073, Germany

Supplementary Data S1

Differences between bear species in % of damage reduction (DR)

AMERICAN BLACK BEAR

Protection of beehives was much more effective (median DR = 97.1%, 95% CI = 80.2-100%) than that of neighbourhood safety (10.7%, 0.0-51.5%; U = 10.5, p = 0.001), but the difference between other assets was insignificant (U from 6.0 to 19.5, p from 0.061 to 0.185).

Husbandry was much more effective (94.2%, 66.7-100%) than aversion (10.7%, -13.9 to 68.0%; U = 13.0, p = 0.003) and non-invasive management (27.6%, 0-79.7%; U = 15.0, p = 0.013). The difference between other intervention categories was insignificant (U from 14.5 to 52.5, p from 0.061 to 0.620).

Electric fences were significantly more effective (100%, 93.8-100%) than mixed deterrents (10.7%, 0-20.0%; U = 0.0, p = 0.013) and translocation (74.4%, 57.1-92.0%; U = 2.0, p = 0.017) and marginally more effective than calving control (100%), change of habits (-3.4%, -11.1% to 0%) and vegetation care (0.25%) pooled (U = 3.5, p = 0.052). Translocation (74.4%, 57.1-92.0%) was significantly more effective than mixed deterrents (10.7%, 0-20.0%; U = 0.0, p = 0.011). The difference between other intervention types was insignificant (U from 3.5 to 8.5, p from 0.096 to 1.000).

Excluded for small sample size (n ≤ 3): cattle, mixed assets and tree plantations (assets), lethal control (intervention categories), acoustic deterrents, chemical deterrents, enclosure, food/garbage isolation, guarding animals, shooting and supplemental feeding (intervention types).

BROWN BEAR

No difference was found in DR across the assets (U from 7.5 to 20.0, p from 0.610 to 1.000) and intervention categories (U from 8.0 to 15.5, p from 0.143 to 0.624).

Excluded for small sample size (n ≤ 3): mixed assets (assets), lethal control (intervention categories) and all intervention types.

ASIATIC BLACK BEAR, POLAR BEAR AND SUN BEAR

No comparisons of DR were done within these species due to small sample size (n = 8 for polar bear, n = 4 for Asiatic black bear and n = 1 for sun bear).
