# Supplementary material for: Variation and conservation implications of the effectiveness of anti-bear interventions
Source: Sci Rep. 2020 Sep 18;10:15341. doi: 10.1038/s41598-020-72343-6 (PMC7501236; doi:10.1038/s41598-020-72343-6)
Supplement: Supplementary file 2 [file 41598_2020_72343_MOESM2_ESM.pdf]

Variation and conservation implications of the effectiveness of anti-bear interventions

Igor Khorozyan, Matthias Waltert

Department of Conservation Biology, Georg-August-Universität Göttingen, Bürgerstr. 50, Göttingen 37073, Germany

Supplementary Data S2

Differences between bear densities among bear species and countries

Densities of American black bears (23.6 ± 2.5 ind./100 km^2) and Asiatic black bears (17.8 ± 3.9 ind./100 km^2) were much higher than those of brown bears (4.3 ± 1.6 ind./100 km^2; Mann-Whitney U from 6.0 to 74.0, p from < 0.001 to 0.006) and polar bears (0.2 ± 0.01 ind./100 km^2; U = 0.0, p from < 0.001 to 0.003). Brown bear densities were significantly higher than polar bear densities (U = 14.0, p < 0.001). Bear densities were higher in the USA and Japan than in Canada and Norway (U from 0.0 to 143.0, p from 0.001 to 0.016).
